# Insights into the Role of Bioactive Food Ingredients and the Microbiome in Idiopathic Pulmonary Fibrosis

**DOI:** 10.3390/ijms21176051

**Published:** 2020-08-22

**Authors:** Josep Mercader-Barceló, Joan Truyols-Vives, Carlos Río, Nora López-Safont, Ernest Sala-Llinàs, Alice Chaplin

**Affiliations:** 1 Inflammation, Repair and Cancer in Respiratory Diseases Research Group, Balearic Islands Health Research Institute (IdISBa), Ctra. Valldemossa 79, 07120 Palma, Balearic Islands, Spain; carlos.rio@ssib.es (C.R.); ernest.sala@ssib.es (E.S.-L.); 2 Department of Fundamental Biology and Health Sciences, University of the Balearic Islands (UIB), 07122 Palma, Balearic Islands, Spain; joantruyolsvives@gmail.com; 3 Foners Medicina Veterinària i Innovació SLP, Foners 27, 07006 Palma, Balearic Islands, Spain; 4 ADEMA School University, University of the Balearic Islands (UIB), 07009 Palma, Balearic Islands, Spain; nlopez@ademaescuelauniversitaria.com; 5 Pneumology Service, Hospital Universitari Son Espases, 07010 Palma de Mallorca, Spain; 6 Centro de Investigación en Red de Enfermedades Respiratorias (CIBERES), 28029 Madrid, Spain; 7 Lipids in Human Pathology Research Group, Balearic Islands Health Research Institute (IdISBa), Ctra. Valldemossa 79, 07120 Palma, Balearic Islands, Spain

**Keywords:** idiopathic pulmonary fibrosis, diet, microbiota, gut-lung axis

## Abstract

Idiopathic pulmonary fibrosis (IPF) is a chronic disease mainly associated with aging and, to date, its causes are still largely unknown. It has been shown that dietary habits can accelerate or delay the occurrence of aging-related diseases; however, their potential role in IPF development has been underestimated so far. The present review summarizes the evidence regarding the relationship between diet and IPF in humans, and in animal models of pulmonary fibrosis, in which we discuss the bioactivity of specific dietary food ingredients, including fatty acids, peptides, amino acids, carbohydrates, vitamins, minerals and phytochemicals. Interestingly, many animal studies reveal preventive and therapeutic effects of particular compounds. Furthermore, it has been recently suggested that the lung and gut microbiota could be involved in IPF, a relationship which may be linked to changes in immunological and inflammatory factors. Thus, all the evidence so far puts forward the idea that the gut-lung axis could be modulated by dietary factors, which in turn have an influence on IPF development. Overall, the data reviewed here support the notion of identifying food ingredients with potential benefits in IPF, with the ultimate aim of designing nutritional approaches as an adjuvant therapeutic strategy.

## 1. Introduction

Idiopathic pulmonary fibrosis (IPF) is a chronic, progressive, irreversible and heterogeneous disease characterised by the excessive deposition of extracellular matrix associated with progressive decline in lung function and respiratory failure [1]. IPF occurs primarily in older adults with a median survival of 2–3 years from diagnosis and, due to an increase in its incidence, it is becoming an economic burden on global health care [2]. The histopathological hallmark used for diagnosis is the occurrence of a usual interstitial pneumonia (UIP) pattern [2]. The current treatment options that show a modest benefit are the anti-fibrotic drugs nintedanib and pirfenidone; however, there is still no cure for IPF [1,2]. Current paradigms suggest that IPF is the consequence of an aberrant repair process, in response to subclinical epithelial injury superimposed on accelerated epithelial aging [2]. Host factors associated to the development of this condition include chronological age, the occurrence of the hallmarks of aging [3,4,5,6], such as telomere attrition, senescence, stem cell exhaustion and mitochondrial dysfunction, and genetic predisposition [7]. Furthermore, environmental factors, including exposure to wood and metal dust, pollution, tobacco smoking, gastric aspiration and viral infection, are thought to play a causative role in the initiation and progression of the disease [2]. Even though it is well known that diet has an influence on the risk of age-related diseases, the potential role of dietary factors has not been considered in this complex scenario. However, there is an increasing number of evidences in animal models suggesting a role for specific bioactive food ingredients in the promotion and mitigation of pulmonary fibrosis. Moreover, alterations in glucose and lipid metabolism are present in pulmonary fibrosis [8,9], and metabolic reprogramming in IPF patients has been described [10]. On the other hand, it has been recently suggested that the microbiome could actually influence the risk of initiation and/or progression of IPF [11,12,13], where gut and airway microbiota are considered relevant players. Here, we will review the studies carried out to date which investigate the role of bioactive food ingredients and microbiota in IPF, and discuss how their potential interaction could have an effect on the development and mitigation of this disease.

## 2. Pathogenesis of IPF

The UIP pattern characteristic of IPF includes temporal and spatially heterogeneous fibrosis, advanced scarring and microscopic honeycombing, clusters of fibroblasts and myofibroblasts (fibroblastic foci), the accumulation of hyperplastic type II alveolar epithelial cells, the reduction of type I alveolar epithelial cells and exaggerated deposition of disorganised collagen and extracellular matrix (ECM), resulting in the distortion of normal lung architecture [2]. In this context, IPF is thought to be the consequence of an aberrant repair process, in response to complex interactions between host and environmental factors. Host factors include genetic and epigenetic features that contribute to the development of an inherently susceptible and dysfunctional epithelium, to recurrent micro-injuries from cigarette smoke, wood and metal dust, gastro-oesophageal reflux, and viral infection [1]. The genetic predisposition to develop IPF has been shown in carriers of variants of genes that affect lung epithelial cells [2]. Certain epigenetic changes have also been identified in IPF patients [14], but the triggering environmental agents and the mechanisms involved in the epigenetic alterations remain elusive. The genetically and/or epigenetically predisposed epithelium displays a limited regenerative ability following recurrent injury, which is crucial for the propagation of IPF.

As a response to sustained injury, epithelial cells acquire features of mesenchymal cells, a process termed epithelial-mesenchymal transition (EMT), which is initiated by the transforming growth factor β1 (TGF-β1) [7,15]. These reprogrammed cells express α smooth muscle actin (αSMA), type I collagen and N-cadherin, and these changes trigger the loss of their polarity and tight junctions, and become more mobile [15]. Furthermore, the alveolar epithelial basement membrane is disrupted as a consequence to the damaged and dysfunctional epithelium, which favours the leakage of fibrin and fibronectin into the interstitial and alveolar spaces, as well as the intrusion of inflammatory cells and the migration of mesenchymal cells [7].

Activated epithelial and endothelial cells promote an aberrant epithelial-mesenchymal crosstalk [7,15]. Circulating fibrocytes are recruited by the alveolar epithelium, differentiate into fibroblasts or myofibroblasts, and produce collagen and cytokines that induce collagen deposition [7]. Epithelial cells release TGF-β1, which leads to the differentiation of resident pulmonary fibroblasts into myofibroblasts that secrete excessive amounts of matrix, platelet-derived growth factor (PDGF), which increases fibroblast proliferation, and Wnt proteins, that stimulate fibroblasts to produce type I collagen [7,15]. Chronically activated fibroblasts acquire an invasive phenotype, and are resistant to apoptosis, which leads to their retention in IPF lungs, allowing the fibroblasts to persistently deposit collagen [7,15]. In addition to abnormal quantities of matrix proteins, ECM remodelling is characterised by its stiffness and altered composition, which seem to stimulate the production of more collagen. ECM changes include an increase in the levels of matrix metalloproteases (MMP), such as MMP-1 and MMP-7, which are involved in ECM degradation; lysyl oxidase-like proteins, causing collagen accumulation and deposition; periostin, which promotes mesenchymal cell proliferation and fibrosis; and osteopontin, a proinflammatory cytokine involved in tissue repair [16].

Both oxidative stress and immunological factors may play a pathogenetic role in IPF [17,18]. An imbalance between oxidants and antioxidants is observed in IPF patients, who show elevated concentrations of oxidants, depleted glutathione levels, and reduced activity of the nuclear factor-erythroid-related factor 2 (Nrf-2), the antioxidant enzyme haem oxygenase 1, and superoxide dismutase (SOD) [17,18]. Furthermore, immune function is also altered in IPF, whereby changes in the innate immune response to infection and tissue injury have been observed, as well as in adaptive immunity, highlighted by the activation of CD4+ T-cells and a decrease in levels of T regulatory cells (Tregs) [16].

### Contribution of Aging Hallmarks to IPF

Aging is the most evident host factor that influences IPF occurrence. The main molecular and cellular hallmarks of aging are present in IPF, which include telomere attrition, genomic instability, mitochondrial dysfunction, epigenetic alterations, cellular senescence, loss of proteostasis, dysregulated nutrient sensing, stem cell exhaustion and altered intercellular communication [19]. Furthermore, it has been shown that some of these hallmarks mediate the development of the disease [3,4,6,20]. For example, abnormal telomere shortening due to mutations in telomerase-related genes is linked to poor prognosis [5]. Aging-associated mitochondrial changes have also been identified in IPF, including an imbalance in mitochondrial dynamics, increased mitochondrial reactive oxygen species (ROS) levels and DNA mutations, leading to decreased mitochondrial respiration and ATP production. Collectively, these changes contribute to the senescent phenotype in lung fibroblasts [2,3,20]. Higher levels of senescence-associated cell cycle repressors, p21, p16 and/or p53 and beta-galactosidase activity, are found in lung cells from IPF patients, and the contributing role of senescence in IPF has been demonstrated with the administration of a senolytic drug [6]. Furthermore, bone marrow stem cells from IPF patients showed decreased stemness and capacity to prevent lung fibrosis progression [4].

However, the development of pulmonary fibrosis in individuals with no identifiable mutations, or that have not been exposed to the abovementioned environmental inputs, means that other factors must be considered. Since it is well known that diet influences the risk of age-related diseases, and that the microbiome is highly implicated in a wide range of conditions, we decided to delve into the data published so far, in order to elucidate their potential involvement in IPF.

## 3. The Role of Nutritional Factors in IPF

### 3.1. Nutrition and Aging

Nutrition is intimately linked to health and disease. Numerous epidemiological studies support the premise that unhealthy dietary habits increase the risk of age-related chronic diseases and accelerate mortality [21]. On the other hand, healthy dietary patterns could delay or prevent the occurrence of age-related diseases, thus contributing to extend a person’s health and lifespan [22]. Accordingly, specific nutritional recommendations for particular subpopulations are associated with the prevention of age-related diseases; for instance, the use of calcium supplementation in post-menopausal women to reduce osteoporosis risk [23]. In addition to exerting a preventive role, the beneficial effect of particular nutrients on aging diseases is also shown in the elderly, as it happens with the intake of leucine in individuals with sarcopenia, in order to reduce muscle mass loss [24]. Furthermore, the adherence to healthy dietary patterns, such as plant-based diets, may increase overall survival in elderly individuals [25]. These data highlight the importance of precision nutrition in relation to aging, and point towards the need to design personalized evidence-based nutritional programmes to this end.

The best-known nutritional strategy to increase longevity in several animal species is the reduction of food intake without reaching malnutrition. Dietary restriction exerts a protective effect against obesity, type 2 diabetes, inflammation, hypertension and cardiovascular disease, and reduces the metabolic risk factors associated with cancer [26]. A lower intake of particular nutrients seems to be the key point for ameliorating aging, rather than calorie restriction [27]. For example, reducing protein or specific amino acids in the diet, such as methionine and tryptophan, induces protective mechanisms and extends average and maximal lifespan in experimental animals. The search of bioactive compounds able to mimic the effects of dietary restriction is of interest, given the difficulty to follow dietary restriction, particularly in the elderly. Supplementation with resveratrol increased the health and lifespan in mice, by improving mitochondrial function through the activation of sirtuin 1 (SIRT1) [28,29]. This discovery sparked an interest in molecules with an anti-aging potential, such as curcumin, epigallocatechin gallate, quercetin and resveratrol [30], which are naturally occurring, and have since demonstrated anti-aging properties in clinical trials.

However, the effects of dietary manipulations on respiratory function and age-related respiratory chronic diseases have been hardly investigated. Hegab et al. [31] recently reported that calorie restriction reversed the decrease in lung stem cells, the number and function of mitochondria, and lung inflammatory cell infiltration in old mice. Moreover, the same authors demonstrated that exposure to a high-fat diet exacerbated aging-induced lung inflammation and mitochondrial deterioration; effects that could be reversed by switching to a low-fat diet [32]. Moreover, maternal exposure to a high-fat diet negatively affected offspring lung function, and seemed to increase their susceptibility to lung diseases later in life [33]. Altogether, these results suggest that certain dietary habits may improve aging-related respiratory function.

### 3.2. Bioactive Food Ingredients Influencing IPF

Up until now, most studies on IPF have mainly focused on factors that can damage the airway. So far, the role of nutritional status in the development of IPF has been poorly evaluated in humans and, to the best of our knowledge, few epidemiological studies have analysed diet in IPF. Two case-control studies in a Japanese population found a lower fruit and higher meat consumption, together with a higher intake of saturated fat, in IPF patients [34,35]. Dietary lipid composition may contribute to the alterations observed in pulmonary surfactant phospholipids of IPF patients, whereby a lower content in polyunsaturated fatty acids at the expense of saturated fatty acids may account for their impaired biophysical surfactant function [36]. Dietary factors may influence both the risk of IPF occurrence and disease progression. On the other hand, it is also thought that IPF could affect nutritional status, since IPF patients present a high prevalence of malnutrition [37], evidencing the need for specific nutritional evaluation and counselling [38].

Interestingly, among IPF patients, cases of cardiovascular diseases, obesity, diabetes mellitus and dyslipidaemia are frequently reported [39]. Such associations suggest that dietary factors commonly involved in these metabolic diseases may also participate in the aetiology of pulmonary fibrosis, and the evidence demonstrated in experimental studies supports this rationale. In mice, neonatal overfeeding induces obesity in adulthood, and worsens airway hyperresponsiveness to methacholine, highlighted by higher amounts of collagen accumulated surrounding the bronchi and lung TGF-β1 expression [40]. Such hyperresponsiveness is also observed when adult mice are exposed to a high-fat diet [41]. The higher circulating insulin level exhibited by adult mice fed a high-fat diet seems to be the agent that enhances TGF-β1 expression in the bronchial epithelium, pointing to insulin resistance as an important player in the development of lung fibrosis. Notably, exposure to a hypercaloric diet triggers pulmonary fibrosis without the involvement of any other intentional external agent [42,43], which strengthens the hypothesis that particular bioactive food ingredients could play a causative role in the development of IPF. These observations prompted us to review the evidence regarding the participation of macronutrients, micronutrients and other bioactive food ingredients, either in the occurrence or in the attenuation of pulmonary fibrosis.

#### 3.2.1. Macronutrients

##### Lipids

The exposure to diets rich in lipids triggers the occurrence of pulmonary fibrosis per se [42,43] and worsens the airway responsiveness to a challenging agent [40,41], supporting a potential role for dietary lipids as a direct causative agent of pulmonary fibrosis. The involvement of dietary lipids and lipid handling is reinforced by the fact that alterations in enzymes involved in lipid metabolism exacerbates pulmonary fibrosis [8], and that IPF patients present a decreased sphingolipid metabolism and mitochondrial β-oxidation capacity [44]. Interestingly, a recent study revealed a specific circulating lipid profile in IPF patients, and identified particular lipids that could be used as biomarkers to monitor the disease or provide prognostic information [45].

However, the effect of dietary lipids could depend on the type of fatty acid or the lipid source. Accordingly, one of the first experiments addressing these issues revealed that the intake of coconut oil or beef tallow as lipid sources triggered lower increases in lung hydroxyproline content and lipid peroxidation after the administration of bleomycin (a DNA-interactive antitumor agent commonly used to induce pulmonary fibrosis), indicating that alterations in dietary lipids can, indeed, reduce the severity of pulmonary fibrosis [46]. Here, we aim to analyse the updated evidence on the effects of different types of fatty acids on pulmonary fibrosis:

##### Saturated Fatty Acids (SFA)

Exposure to a high-fat diet has been shown to trigger the occurrence of incipient lung fibrosis [43], whereby the authors of this investigation noted that an increase in neutrophils in the pulmonary parenchyma may play a significant role in the development of lung fibrosis. Furthermore, in another experiment in which lung fibrosis was induced by a high-fat diet, it was observed that the levels of TGF-β1 in airway epithelial cells were increased, which was accompanied by an increased collagen deposition and expression of profibrotic factors [42], which suggest a causative role of a high intake of fatty acids in EMT. Although such a causative role could not be solely attributed to SFA in the previous experiments, Chu SG et al. [47] have recently demonstrated that the exposure to a high-fat diet rich in palmitic acid increases pulmonary fibrosis after bleomycin administration in wild-type mice. This effect was linked to the activation of the unfolded protein response and apoptosis in lung epithelial cells [47]. Moreover, these authors demonstrated the causative role of lipotoxicity on lung fibrosis by inhibiting the fatty acid transporter CD36. These results are in accordance with the higher relative contents of palmitic acid found in pulmonary surfactant phospholipids of IPF patients [36] and the positive correlation between SFA intake and IPF in a Japanese population [35], thus suggesting that a high SFA intake might increase the risk of IPF.

##### Polyunsaturated Fatty Acids (PUFA)

Following the observation that IPF patients show lower relative contents of oleic acid surfactant phospholipids [36], it has been hypothesized that a high PUFA intake could mitigate IPF. This question has been addressed in in vivo studies, showing that the intake of fish oil, rich in eicosapentaenoic acid (20:5, ω-3), reduces lung protein and hydroxyproline contents after a bleomycin challenge [48]. In the context of lung fibrosis, a relevant example of the importance of dietary lipids in the perinatal period for the development of diseases in adulthood comes from an experiment in which maternal diet supplementation with docosahexaenoic acid attenuated pulmonary fibrosis and improved lung function in mouse pups exposed to perinatal inflammation [49]. Furthermore, the mitigation of lung fibrosis has been demonstrated with long-chain ω-3 PUFAs (docosahexaenoic acid (22:6, ω-3)) [50], short-chain ω-3 PUFA present in flaxseed oil [51,52], and with ω-6 PUFAs, such as γ-linolenic acid (18:3, ω-6) [53]. The effects attributed to the anti-fibrotic properties of PUFA could be mediated, in part, through their conversion to resolvinD_1_, which inhibits EMT in human alveolar type II cells [54], and through peroxisome proliferator-activated receptor γ (PPARγ). PPARγ agonists exhibit anti-fibrotic activity, and nitrated fatty acids, which are produced when unsaturated fatty acids react with nitric oxide (NO), are potent PPARγ agonists. Interestingly, the treatment with nitrated fatty acids reversed myofibroblast differentiation and reduced collagen deposition once lung fibrosis was established, suggesting a therapeutic role for nitrated fatty acids [55]. In all these experiments, PUFA was provided before pulmonary fibrosis was induced and, collectively, the results indicate that an adequate dietary PUFA intake might reduce the risk of lung fibrosis development.

##### Carbohydrates

The addition of fructose to a high-fat diet enhanced the development of pulmonary fibrosis without the participation of another intentional agent in mice [43], suggesting that high dietary contents of greatly available carbohydrates, both mono and disaccharides, might be associated with an increased risk of IPF. In fact, GLUT1-dependent glycolysis is increased in aged lungs and activates lung fibroblasts and lung fibrogenesis [9]. However, most of the studies that analyse the role of dietary carbohydrates in experimental pulmonary fibrosis attempt to mitigate fibrosis with carbohydrates, particularly specific polysaccharides, providing interesting results. For instance, barley-extracted β-glucans, which are long-chain polymers of glucose, improved hydroxyproline and oxidative stress markers, when given either in combination with N-acetylcysteine before bleomycin instillation, or alone after bleomycin administration [56]. The beneficial effects of β-glucans may rely on their ability to stimulate the immune system and antioxidant activity. Similarly, an enhanced antioxidant activity and/or immune system response also seems to explain the attenuation of pulmonary fibrosis elicited by the administration of polysaccharides from the fungi *Ophiocordyceps lanpingensis* [57] and *Ganoderma lucidum* [58]. Other polysaccharides of interest include fucoidan, a sulphated polysaccharide found in brown seaweed [59], and chitosan, a linear polysaccharide with antibacterial, antifungal, antioxidant and anti-inflammatory activities. Both these polysaccharides have shown to exert anti-fibrotic effects when administrated orally, before and after lung damage [60,61]. Anti-fibrotic effects linked to EMT inhibition were also described by the administration of plant-derived polysaccharides, including those extracted from *Dendrobium officionale* [62] or from the ancient Chinese herbal formula Yupingfeng [63].

##### Amino Acids, Amino Acid Derivatives and Peptides

IPF patients show an alteration in amino acid metabolism, characterised by higher amounts of proline, 4-hydroxyproline, alanine, valine, leucine, isoleucine and allysine, detected in both lung tissue and breath [64]. This has led to the question of whether the supply of functional amino acids could represent a true therapeutic strategy.

In vivo studies have addressed this issue and, in fact, oral treatment with L-arginine improved arginine metabolism in bleomycin-treated mice and reduced lung damage [65]. Similarly, in radiation-induced lung fibrosis, L-arginine reduced hydroxyproline content, procollagen I and III expression and angiotensin converting enzyme activity, which was attributed to the exogenous NO supply [66]. Likewise, pre-treatment with aerosolized arginine in experimental models of LPS-induced fibrosis reduced collagen deposition, apoptosis of alveolar cells and the expression of inflammatory cytokines and chemokines [67]. The immunomodulatory ability of certain amino acids, including L-arginine, glycine and L-norvaline, a valine isomer, seem to explain, at least in part, the protection against pulmonary fibrosis, since a reduction in the accumulation of neutrophils and macrophages and a restoration in peripheral blood Tregs were observed in animal models of lung fibrosis treated with these amino acids [65,67].

Taurine is a cysteine-derived amino acid that naturally occurs in the body. Interestingly, its anti-fibrotic potential has been documented in animal models of pulmonary fibrosis, which is explained, in part, by its antioxidant and immunomodulatory abilities [68,69]. In hamsters, diet supplementation with taurine reduced lung collagen, lipid peroxidation and SOD activity [68], and the administration of taurine both before and after bleomycin instillation reduced the amount of neutrophils in the bronchoalveolar lavage fluid (BALF) [69]. In a considerable number of experiments, the anti-fibrotic potential of taurine was studied, together with niacin, a vitamin B3 form, that when given alone, also attenuates pulmonary fibrosis [68,70], and this co-treatment exhibited a potent anti-fibrotic effect [71,72].

Recently, it was demonstrated that a small peptide isolated from the edible seaweed *Eucheuma*, named EZY-1, exhibited anti-fibrotic properties, with a superior potency and safety than pirfenidone [73], by altering the binding to PDGF receptor β and the TGF-β/Smad signal transduction. Another small peptide showing anti-fibrotic activity is the metal chelating tripeptide GHK, a naturally occurring human plasma component, the levels of which reflect regenerative capacity, and which regulates wound healing processes by inhibiting TGF-β secretion [74]. The administration of GHK inhibited collagen deposition and the inflammatory response, and suppressed EMT [75].

#### 3.2.2. Micronutrients

##### Vitamins

The levels of vitamers with antioxidant activity are increased in the BALF of IPF patients, in an attempt to restore oxidative balance [76]. Such vitamers include retinol, ascorbic acid and α-tocopherol. On the contrary, these non-enzymatic antioxidants are reduced in the bleomycin model of lung fibrosis [77], highlighting the discrepancy between the bleomycin model and the human disease.

(1) Vitamin A

Retinoic acid, the bioactive metabolite of vitamin A, is an important signalling molecule during normal early lung development, and has anti-fibrotic and anti-inflammatory properties [78]. All-trans retinoic acid treatment has shown to ameliorate irradiation and bleomycin-induced lung fibrosis in mice, and its anti-fibrotic mechanisms include the decrease in TGF-β, Il-6 and Il-17A cytokine production, and a shift in the Treg/Th17 ratio [79].

(2) Vitamin B

Niacin is a vitamin B3 form involved in DNA repair, as a precursor of the coenzymes nicotinamide adenine dinucleotide and nicotinamide adenine dinucleotide phosphate. As such, its ability to enhance DNA repair caused by bleomycin has been demonstrated, being one of the first described agents able to attenuate bleomycin-induced lung fibrosis [70]. Subsequently, many studies have analysed the combination of niacin with taurine, which is expected to act through different mechanisms [68,72].

(3) Vitamin C

Vitamin C is involved in tissue repair and collagen secretion, and its deficiency leads to impaired collagen synthesis. In pulmonary fibrosis induced by chromium, paraquat or LPS, the damage was reduced by the subsequent or simultaneous administration of vitamin C [80,81,82]. The mechanisms of action of vitamin C, reported in the paraquat model, included reduced neutrophil recruitment and IL-17 and TGF-β secretion, and increased SOD and catalase levels [81]. Intriguingly, even though these results suggest the potential therapeutic role of vitamin C, another study reported that the depletion of ascorbate in the diet inhibits the increased neutrophilic incursion and lavage protein concentration induced by silica instillation, which may reflect the pro-oxidant function for ascorbate or the lack of ascorbate-dependent collagen hydroxylation [83].

(4) Vitamin D

Serum vitamin D has been proposed as a biomarker of IPF prognosis [84]. IPF patients show deficient circulating vitamin D levels, which correlate with disease prognosis and all-cause mortality [84,85]. Furthermore, vitamin D deficiency and bone fragility have been associated with IPF [86]. In light of this, studies have shown a potential therapeutic role for vitamin D, such as the anti-fibrotic effect of an early vitamin D supplementation in the bleomycin model of lung fibrosis [87], and in paraquat-treated mice after a fibrotic challenge [88].

(5) Vitamin E

The role of vitamin E in the development of pulmonary fibrosis has been shown in both vitamin E deficient animals, which show a more severe interstitial pneumonitis and emphysematous lesions [89], and vitamin E treated animals, in which lung fibrosis was induced by diverse pro-fibrotic agents, including bleomycin [90], actinomycete [91], amioradone [92], radiation [93], chromium [80], and nitrogen mustard melphalan [94]. In these animal models, the mitigation of fibrosis was mainly attributed to the antioxidant activity of vitamin E. In humans, vitamin E was evaluated in combination with pentoxifylline [95], showing that chronic treatment reversed lung damage in patients with radiation-induced fibrosis, thus highlighting the anti-fibrotic potential of vitamin E.

##### Minerals and Salt

(1) Iron

The lungs of IPF patients show numerous aspects of dysfunctional iron metabolism, including increased iron levels, the presence of iron-laden macrophages, and iron-induced oxidative stress [96]. Fibrosis severity and pulmonary hypertension are positively associated with iron deposition in lung tissue [97] and epithelial lining fluid [98]. In BALF isolated cells, iron accumulation increases ROS generation, which is thought to play a role in macrophage activation [99]. A causative role for iron accumulation in IPF is demonstrated in experimental animals, in which fibrosis and lung function decline are prevented by the intranasal administration of an iron chelator [100]. Furthermore, animals maintained on an iron deficient diet do not accumulate lung collagen and do not increase lung lipid peroxidation after lung damage induction [83,101], which strongly supports a potential role of dietary iron in pulmonary fibrosis.

(2) Copper

Excess of copper plays an important role in the aetiology and pathogenesis of many diseases, including IPF [102], and its availability has been proposed as a distinctive factor for the development of lung fibrosis and emphysema [103]. Copper is essential for the enzymatic activity of lysyl oxidase (LOX), the cytosolic SOD isoform, and amine oxidase copper containing-3 (AOC3), proteins which are known to have an important role in IPF. LOX regulates the covalent cross-linking of ECM collagen and elastin, whereas AOC3 is a membrane-bound protein that oxidizes biogenic and dietary amines, and activates leukocyte extravasation [104]. The role of AOC3 in pulmonary fibrosis development has been shown using AOC3-deficient mice, which were protected from lung fibrosis, as well as in wild-type mice treated with an AOC3 inhibitor [105]. The reduction of copper levels achieved by a low-copper diet and the administration of a chelator agent regressed the overexpression of collagen-I, LOX, MMP2, MMP8 and TIMP1 in the lungs of fibrotic mice [106], suggesting that the management of dietary copper might influence pulmonary fibrosis development.

(3) Sodium Chloride

It has been suggested that dietary salt may influence IPF, based on the fact that its intake is associated with cardiac and renal fibrosis and, in cultured cells, promotes the trans-differentiation of monocytes into fibrocytes [107,108]. Despite the fact that a high salt diet did not exacerbate lung fibrosis after bleomycin administration in mice, a low salt diet attenuated fibrosis, which was linked to a normalization in leukocyte number and a reduction in fibrocyte number [109].

#### 3.2.3. Phytochemicals

An increasing number of studies have analysed the anti-fibrotic effect of various phytochemicals in animal models of lung fibrosis. Quercetin, curcumin, resveratrol, epigallocatechin-3-gallate, S-allyl compounds and several lignans are the most studied phytochemicals in the context of lung fibrosis. Studies in which phytochemicals were orally administrated or supplemented in the diet are included in Table 1, together with the proposed anti-fibrotic molecular mechanisms, which involve anti-oxidant, anti-inflammatory and inhibitory effects on EMT.

##### Quercetin

The flavonoid quercetin is found in vegetables and fruits, particularly in onions, apples and broccoli. Quercetin presents anti-aging properties, due to its antioxidant activity and ability to induce apoptosis in senescent cells, thereby mitigating the severity of several age-related chronic diseases [110]. In experimental pulmonary fibrosis, the anti-fibrotic effect of quercetin was shown to be dependent on the activation of Nrf2-responsive genes, using Nrf2 deficient mice [111]. In addition to its antioxidant and anti-inflammatory activities, it is involved in the reversal of the resistance to apoptosis, and the reduction of the expression of senescence markers p21 and p19-ARF, and the senescence-associated secretory phenotype, which are observed in the lungs of bleomycin-treated aged mice [112]. Importantly, the removal of senescent cells with dasatinib and quercetin ameliorated pulmonary function in experimental animals [6]. This treatment was also assayed in a human clinical trial, demonstrating that IPF patients improved physical function [113], highlighting the relevant role that a natural bioactive compound such as quercetin could exert in the management of IPF.

##### Curcumin

The flavonoid curcumin is present in turmeric (*Curcuma longa*), and is known for its antioxidant and anti-inflammatory effects mediated by its ability to inhibit NF-kB. Curcumin arises as a potential anti-aging molecule [30] and its use against pulmonary diseases has been posed [114]. In experimental lung fibrosis, curcumin administration after lung damage induction ameliorates oxidative stress and inflammation-related parameters, changes that are accompanied by signs of reduced fibrosis [115,116,117]. Beneficial effects are also reported when dietary curcumin is provided before the induction of fibrosis, including the enhancement of antioxidant defenses, amelioration of fibrosis and survival improvement [118]. In addition to the enhancement of antioxidant and anti-inflammatory activities, experiments in cultured lung fibroblasts reveal curcumin’s ability to decrease fibroblast proliferation and migration, increase apoptotic markers, and stimulate proteins involved in matrix degradation [119]. However, despite its potential, curcumin activity is limited by its poor bioavailability, and this feature could explain why an intraperitoneal administration exerts superior anti-fibrotic effects than oral treatment [120]. To circumvent this, novel strategies of curcumin administration are being developed. Hu Y. et al. [121] developed inhalable curcumin-loaded poly(lactic-co-glycolic) acid large porous microparticles, and showed that they entered into the lung tissue and triggered striking anti-fibrotic effects [121], serving as a potential therapy applied to IPF.

##### Resveratrol

Resveratrol is a stilbenoid polyphenol present in grapes, known for its antioxidant activity, its ability to enhance mitochondrial biogenesis through the activation of PPARγ coactivator-1α (PGC-1α), its ability to activate serine/threonine kinase AMP-activated protein kinase complex (AMPK) signalling, and its capacity to extend lifespan in several species, including mammals, by activating SIRT1 [28,29]. In animal models of lung fibrosis, the anti-fibrotic potential of resveratrol has been analysed, showing promising results. Resveratrol reduces collagen content when it is administered after a fibrotic challenge, a change accompanied by a reduction in markers of oxidative stress and inflammation [122,123]. Moreover, resveratrol attenuates EMT by directly acting on IPF fibroblasts, and inhibiting TGF-β-induced proliferation and differentiation into myofibroblasts [124]. SIRT1 activation and other mechanisms of action have been described for the anti-fibrotic effect of resveratrol, which are detailed in Table 1 [125,126,127].

##### Flaxseed Lignans and Schisandrin B

Plant lignans are fibre-associated polyphenolic compounds that form lignin, a constituent of the plant cell wall. They are particularly abundant in flaxseed, rye bran and oat bran, and are known for their antioxidant properties. The supplementation of the diet with flaxseeds triggers anti-fibrotic effects, both before and after lung damage [128,129], doubling the survival rate when analysed as a therapeutic agent. When flaxseeds are given as oil, this treatment also reduces lung damage, as previously mentioned, an effect that is attributed to their high short-chain ω-3 PUFA content [51,52]. However, it has been demonstrated that lignans from wholegrain flaxseed decrease inflammation, lung injury and eventual fibrosis while it improves survival after radiation exposure, evidencing that it is a bioactive component responsible for lung damage mitigation [130]. Schisandrin B, a highly abundant lignan present in *Schisandra chinensis*, known for its diverse beneficial health effects, was also shown to attenuate pulmonary fibrosis in experimental animals [131,132].

##### Epigallocatechin-3-Gallate

The polyphenol epigallocatechin-3-gallate (EGCG) is a major component of green tea, which aroused interest due to its anti-aging and immunomodulatory properties [133]. The administration of green tea extract reduces lung fibrosis in vivo, which is attributed to its anti-inflammatory and antioxidant activities [77,134]. Moreover, a direct inhibitory fibroblast activation is also described for EGCG [135].

##### S-allyl-Compounds

Garlic possesses antioxidant activity, and has been traditionally used for the treatment of ailments associated with aging. Garlic bioactive compounds include S-allyl-cysteine and S-allylmercaptocysteine, organosulphur compounds that target the Nrf2 and NF-kB pathways, which are involved in the in vivo anti-inflammatory and anti-fibrotic activities [136,137,138,139,140]. Notably, S-allyl compounds might not only act as therapeutic, but also as preventive, agents [138,140]. Importantly, the beneficial effect of S-allyl-compounds might be achievable from diet, since the induced damage in the lung was alleviated by the supplementation of the diet with S-allyl cysteine or the administration of a water soluble aged garlic [139]. Compared to the well-known anti-fibrotic effect of N-acetylcysteine, those elicited by S-allyl-compounds were higher, which highlight the interest of S-allyl compounds as potential dietary agents to reduce pulmonary fibrosis development.

**Table 1:** Effects of phytochemicals in animal models of pulmonary fibrosis.

Phytochemical Compound	Dosage	Animal Models	Main Outcomes Related to Oxidative Stress, Inflammation, EMT and Fibrosis
Quercetin [111,141,142,143]	10–100 mg/kg bw/day 800 mg/kg in diet	BLM and amiodarone-induced female and male mice and rats	↓ MDA levels; ↑ Nrf2, CAT and SOD levels
			↓ TNFα, iNOS, IL-13/6, PDGF-β, levels; ↓ H&E staining; ↓ inflammatory cells; ↑ IFN-δ levels
			↓ COL1A2, TGF-β, fibronectin 1, pERK and MMP7 levels; ↓ hydroxyproline content; ↓ Masson’s trichrome staining
Curcumin [115,116,117,118,119,120,144]	74–300mg/kg bw/day 1–5% w/w in diet	Irradiation, paraquat, BLM and amiodarone-induced female and male mice and rats	↓ MPO activity; ↓ TBARS, GST and ROS levels; ↑ cathepsin K and L expression
			↓ NAG, AKP and ACE levels; ↓ c-Jun expression; ↓ TNF-α, superoxide anion and NO release; ↓ mononuclear and PMN cells
			↓ TGF-β1, α-SMA, hydroxyproline, type I collagen expression; ↓ Smad2-3 and ERK1/2 phosphorylation
Resveratrol [122,126,145]	50 and 100 mg/kg bw/2 days 10 mg/kg bw/day 150 mg GSE/kg bw/day	BLM, silica, and particulate matter-induced male rats and mice	↓ MDA levels; ↓ MPO activity; ↑ GSH levels
			↓ IL-6/1-β, TGF-β, TNF- α, NLRP3, ASC and caspase-1 levels; ↓ neutrophils; ↓ H&E staining
			↓ hydroxyproline and collagen content; ↓ Masson’s trichrome staining
Schisandrin B and flaxseed lignans [51,52,128,129,130,131,132]	5–100 mg lignan/kg bw/day 2 mg flaxseed oil/kg bw/day 10–20% lignans, 10% flaxseed (w/w) and 15% flaxseed oil in diet	Irradiation and BLM-induced male and female mice and male rats	↓ MDA, TBARS and Nox4 levels; ↓ nitrotyrosine staining; ↑ CAT and SOD activity
			↓ Alveolar PMN and macrophage influx, ↓ IL-1β/2/4/6/12/17, MIP-1α, VEGF, TNF-α and FGF levels
			↓ TGF-β, MMP7, β-catenin and hydroxyproline levels; ↓ Bax, p21, Smad2 phosphorylation
SAC and SAMC [136,138,139,140]	25–200 mg/kg in bw/day 2 mL AGE/kg bw/2 days 0.15% in diet	TiO_2_, BLM and CCl_4_-induced male rats and male mice	↓ Nox4 and LPO levels; ↑ HO-1, GSH and SOD activity; ↑ Nrf2 and thiol levels
			↓ TNF-α, IL-6 and iNOS levels; ↓ H&E staining; ↓ lymphocyte aggregation
			↓ TGF-β1, ↓ Smad3/P-Smad3, Smad2/P-Smad2 levels; ↓ MMP-9, TIMP-1, α-SMA, fibronectin, collagen 1A1 and collagen III expression; ↓ hydroxyproline content; ↓ Azan-Mallory staining
Astaxanthin [146,147,148]	0.5, 1 and 2 mg/kg bw/day	BLM-induced rats	↑ SOD and CAT activity
			↓ H&E staining
			↓ Hydroxyproline, collagen, vimentin and α-SMA levels; ↓ Masson’s trichrome staining; ↑ E-cadherin levels
Crocin [149,150]	20 and 25 mg/kg bw/day	BLM-induced male rats	↓ MDA and HO-1 levels; ↑ GSH and Nrf2 levels; ↑ GSH-px, SOD and CAT activity
			↓ NO, IL-10, TLR4 and TNF-α levels; ↓ H&E staining; ↓ total inflammatory cell, lymphocyte and neutrophil
			↓ Hydroxyproline content; ↓ Masson’s trichrome staining
Lycopene [151]	5 mg/kg bw/day	BLM-induced male rats	↓ MDA levels; ↑ SOD activity
			↓ H&E staining; NO and TNF-α levels
			↓ Masson’s trichrome staining
Zingerone [152]	50 and 100 mg/kg bw/day	BLM-induced male rats	↓ MDA levels; ↑ SOD and GSH-px activity
			↓ TNFα and IL-1β levels; ↓ H&E and iNOS staining
			↓ TGF-β1 expression; ↓ hydroxyproline content
Ellagic acid [153]	15 mg/kg bw/day	BLM and cyclophosphamide-induced male rats	↓ Lipid peroxidation; ↓ protein oxidation; ↓ NADH oxidize; ↓ MPO activity; ↑ CAT, SOD and GST activity
			↓ NO production
			↓ Hydroxyproline content
Proanthocyanidin [154]	100 mg/kg bw/day	BLM-induced male rats	↓ H&E and iNOS staining; ↓ immune system cells accumulation
			↓ Hydroxyproline content

Studies in which phytochemicals were orally administrated or supplemented in the diet are included. Outcomes are categorized in anti-oxidant, anti-inflammatory and anti-EMT/fibrotic effects. ↑, increase; ↓, decrease; ACE, angiotensin converting enzyme; AGE, aged garlic extract; AKP, alkaline phosphatase; α-SMA, α smooth muscle actin; ASC, apoptosis-associated speck-like protein containing a caspase activation recruitment domain; α-SMA, α-smooth muscle actin; BLM, bleomycin; CAT, catalase; CCl_4_, carbon tetrachloride; COL1A, collagenase 1A; EGCG, epigallocatechin-3-gallate; EMT, epithelial to mesenchymal transition; ERK, extracellular signal-regulated kinase; FGF, fibroblast growth factor; GSE, grape seed extract; GSH, glutathione; GSH-px, glutathione peroxidase; GST, glutathione *S*-transferase; H&E, hematoxylin eosin; HO-1, heme oxygenase-1; IFN-δ, interferon-δ; IL, interleukin; iNOS, inducible nitric oxide synthase; MDA, malondialdehyde; MIP-1α, macrophage inflammatory protein-1α; MMP, matrix metalloproteinase; MPO, myeloperoxidase; NAG, N-acetyl-β-D-glucosaminidase; NLRP3, nucleotide-binding domain and leucine-rich repeat protein 3; NO, nitric oxide; NOX4, NADPH oxidase 4; Nrf2, nuclear factor erythroid 2-related factor 2; PDGF-β, platelet-derived growth factor subunit B; PMN, polimorfonuclear; ROS, reactive oxygen species; SAC, S-allyl-cysteine; SAMC, S-allyl-mercaptocysteine; SOD, superoxide dismutase; TBARS, thiobarbituric acid reactive substances; TGF-β, transforming growth factor-β; TIMP1, tissue inhibitor of metalloproteinase 1; TIO_2_, titanium oxide; TNFα, tumor necrosis factor α; VEGF, vascular endothelial growth factor.

## 4. The Role of Human Microbiota in IPF—An Emerging Therapeutic Strategy

The role of the microbiota, which refers to the microbial (bacterial) community found in the human body, in health and disease is a topic of great interest, and it is now fully established that it is crucial in human wellbeing, and how an imbalance, or dysbiosis, can lead or contribute to a wide range of diseases [155]. In this context, in the last decade, several studies have emerged, showing that lung microbiota plays an important part in airway diseases, including IPF, with a clear connection between lung dysbiosis, mortality and altered immune system pathways. As recently discussed by Dickson et al. [156], the importance of lung microbiota in IPF is evident in the ongoing, current clinical trials, in which, instead of investigating immunosuppression-associated drugs, antibiotics for microbiota manipulation are being trialed.

Historically, the lung has been considered as a sterile organ. However, with the development of non-culture dependent techniques, it has been shown that there is a diverse bacterial microenvironment [157,158]. Even though there is still evidence lacking to establish a solid causal association between altered lung microbiota and IPF progression, data so far are promising, showing that microbial composition and abundance could be predictive of disease progression and are associated to changes in alveolar cytokines and inflammatory pathways. Even though a causal relationship is still not established, it is becoming clearer that lung microbiota is essential for host homeostasis, and could be a focal point of interest in the physiopathology of IPF [159].

In line with this new area of research, it has been observed that patients presenting some pulmonary conditions, including inflammation or impaired lung function, also exhibit gastrointestinal (GIT) diseases. Furthermore, even though the GIT and the respiratory tract present different environments and functions, they have the same embryonic origin and structural similarities, suggesting a potential interaction [160]. Thus, although studies are still scarce, results published to date point towards the role of a gut-lung axis in the pathogenesis of lung diseases [161], with increasing evidence that gut microbiota may be playing a crucial part.

### 4.1. Bacterial Burden, Diversity and Microbial Composition in the Lung in IPF

It has been suggested that three main features regarding lung microbiota are of importance when considering IPF prognosis, pathogenesis and progression: bacterial burden, microbial diversity and overall composition [11].

On one hand, those individuals presenting decreased lung microbial diversity have a worse prognosis, and that an increased bacterial burden at diagnosis is associated with a higher progression of IPF and mortality risk [11,12,13]. Moreover, Molyneaux et al. [12] argue that an abundant, less diverse bacterial community in the lower airways in IPF may be the cause of repetitive alveolar injury, which is thought to be a major factor in its pathogenesis. This was further confirmed by a recent study, in which decreased lung bacterial diversity was associated with increased alveolar concentrations of proinflammatory and profibrotic cytokines and growth factors [11]. More interestingly, a very recent study showed that, even though lung bacterial burden was not associated to IPF severity, it had a strong prognostic significance, by predicting clinical outcomes, providing robust evidence that the lung microbiome could be relevant in IPF prognosis [162].

However, while these data are promising, they do not prove a causal role for lung microbiota in IPF. In order to elucidate this, a range of studies have attempted to determine bacterial composition and how its manipulation can impact IPF pathogenesis. The major bacterial phyla found in lungs is the same as in gut, thus Firmicutes and Bacteroidetes are the predominant bacteria followed by Proteobacteria and Actinobacteria [159]. It has been reported that mice with induced pulmonary fibrosis present lung dysbiosis, with an increase in the Firmicutes phylum and a decrease in Bacteroidetes, for a total of 21 days after injury. This dysbiosis was sustained through all the stages of the fibroproliferative model and chronic injury, and thus supporting the hypothesis that altered pulmonary microbiota can impact injury and repair in IPF [11]. This is in contrast with what was reported later on in a study also carried out in mice, which showed that Bacteroidetes phyla was upregulated in the lung fibrosis model. In particular, Bacteroidacea and Prevotellaceae were the most increased families within this phyla and, considering that most Bacteroidetes bacteria are gram-negative anaerobes, the authors suggest that they are responsible for the lung fibrosis pathogenesis observed in their murine model [163]. Moreover, this same study showed that there was an increase in amino acid, fatty acid and carbohydrate-associated metabolites, which serve as products for Bacteroides and Prevotella growth in the gut. They report an increase in the corresponding metabolic genes in mouse tissues, suggesting that they also promote the growth of the abovementioned phyla in the lung, which in turn promote lung-fibrotic pathogenesis [163].

At the species level, microbial communities differ significantly between the gut and lung. One of the first studies to show an association between specific bacteria or operational taxonomic units (OTUs) and disease progression in IPF reported the presence of *Streptococcus* and *Staphylococcus* in the lungs of patients with a poorer disease outcome, together with *Prevotella* spp., *Veillonella* spp. and *Escherichia* spp. [164] This was further confirmed by other studies, in which *Streptococcus*, *Haemophilus*, *Neisseria* and *Veillonella* spp. have all been observed to be more abundant in patients with IPF (vs. controls) [12,13]. A more recent study demonstrated that the honeycombed lung in IPF patients was associated with alterations in lung microbiota composition, detectable at both the species/genus and family levels of taxonomy, particularly in the *Gemella* spp., which could in turn be altering community composition and promoting injury, due to mucin overexpression and defective mucocillary clearance [165].

Manipulation of lung microbiota has been achieved in other pulmonary diseases by means of antibiotic treatment; this, however, still needs to be determined whether it occurs in IPF [156]. For this, there are ongoing trials of antibiotic treatment in IPF, which will hopefully shed some light on this matter [166,167].

### 4.2. The Gut-Lung Axis: Interplay of the Gut and Lung Microbiota in IPF

Throughout the entire life span, there is a close connection between the bacterial composition of the lung and gut microbiota, leading to the hypothesis of the existence of a host-wide network. This is evidenced by studies showing that changes in a newborn’s diet alter lung microbiota composition, and that fecal transplantation in rats also modifies bacteria in lungs [168,169]. An increasing number of studies are reporting that changes in gut microbiota and its by-products could be having an effect on immune responses and inflammation linked to pulmonary diseases, referred to as the gut-lung axis. Furthermore, it has been reported that microbial by-products, such as endotoxins, metabolites, cytokines and hormones, can make their way to the lung via the bloodstream, thus suggesting a more far-reaching impact than what was previously believed. More interestingly, it seems that this axis can actually be bidirectional, and thus a lung-gut axis also takes place, whereby inflammation in the lung can lead to changes in gut microbiota [161].

In disease, an inflammatory inter-organ cross-talk seems to occur between the lung and the intestine, with a clear association between airway-related diseases and GIT conditions [159]. Even though these two organs share structural similarities and a common embryonic origin, which may account for the resemblances found in disease occurrence, it seems likely that inflammatory and immune-associated components found in these compartments are responsible for the pathological cross-talk observed [170].

To date, the microbiota present in the gut, particularly in the intestine, is the most studied by far. This is not surprising, considering that it is the largest and most diverse community of the human microbiome, with over 4 × 10^13^ microbial cells, which live in a mutualistic relationship with the host and carry out a wide range of functions key to maintain host metabolism and homeostasis of the immune system [171]. In comparison to gut microbiota, the number of bacteria found in other organs in the body is much lower [172].

#### Connecting Lung and Gut Microbiota with The Immune System and Inflammation: An Inter-organ Cross Talk

The impact of gut microbiota on the immune system has been extensively studied, demonstrating so far that bacteria and its by-products interact with the host using pro-inflammatory and regulatory signals, as well as influencing immune cell responses [159]. Currently, an area of great interest is how the gut microbiota can actually influence the immune response and inflammation in the lungs, and vice versa. Thus, interestingly, the connection between the lung microbiota and the immune system is bidirectional, by which major inflammation in the lungs can also have a big impact in lung microbiota composition [173].

The effect of gut microbiota on immunity, and its further impact on lung disease, has been researched in conditions such as COPD, asthma, cystic fibrosis and influenza virus, among others [174]. Moreover, even though data are still scarce, studies have found that the presence of certain bacterial species in the lungs correlate with immune response-associated pathways in the context of IPF, particularly Toll-like receptor signaling [174,175]. In this sense, it has been reported that *Prevotella* and *Staphylococcus* abundance in the lungs of individuals with IPF is negatively correlated with the expression of immune response genes, and that changes in the microbial community structure are associated to changes in the phenotypes of circulating leukocytes [175]. Furthermore, in the absence of lung microbiota, germ-free (GF) mice presented an increased survival rate after (lung) injury exposure [11]. This particular study presented interesting results in this sense, where GF mice were protected from mortality after antibiotic treatment, yet they still exhibited the same severity of pulmonary fibrosis as their conventional counterparts. This observation could be linked to the clinical observation that patients with IPF may not only die from progressive lung fibrosis, but due to diverse inflammatory causes and respiratory infections. Another recent study presents a relationship between specific bacteria, Bacteroides and Prevotella, and fibrotic pathogenesis through IL-17R signaling in a murine model, suggesting that the lung fibrosis occurring in IPF is promoted by specific bacteria through a profibrotic inflammatory cytokine network [163].

However, as with most data regarding host microbiota, discerning the causal relationship between pulmonary and gut microbiota and IPF progression and pathogenesis is still complicated and needs further study, in order to fully understand the mechanistic pathways. It is still unknown whether the impact observed after microbiome manipulation in IPF are due to indirect, off-target effects, mainly on gut microbiota; for this, more studies are required, that can selectively alter lung microbiota without changing gut bacterial composition [156], which is known to greatly interact with the immune system.

## 5. Bioactive Food Ingredients, Microbiota and IPF

The potential roles of bioactive food ingredients and microbiota on IPF may be considered as inter-dependent factors, although their purported role as independent influencing players may also be taken into account. The bioactivity of a given food ingredient cannot be solely exerted by the chemical form it is presented in food, but by a more active by-product derived from gut microbiota. Therefore, its bioactivity in host cells may be dependent on the presence or abundance of particular bacteria capable of metabolizing such compounds. The opposite relationship between food ingredients and gut microbiota could also explain the bioactivity of a compound, in which a nutrient modifies gut bacteria composition, and such changes could determine host health. In this sense, there is accumulating evidence that supports the role of interactions between bioactive food ingredients and gut microbiota, which have been primarily investigated in the context of metabolic diseases [176,177,178].

In the context of chronic lung diseases, the interaction between bioactive food ingredients and gut microbiota, although to a lesser extent, has also been analyzed. Thus, in individuals with cystic fibrosis, the intake of antioxidant vitamins and various flavonoids correlated with the amount of particular gut bacteria, and such variations could potentially influence immune function and inflammation, which are important in cystic fibrosis disease and co-morbidity management [179,180]. These associations pave the way to pose dietary interventions aimed at modifying gut microbiota that will eventually influence respiratory diseases. In line with this idea, a nutritional strategy was designed, whereby mice presenting a pulmonary *Pseudomonas aeruginosa* infection were fed a diet enriched in acidic oligosaccharides derived from pectin [181]. This dietary intervention stimulated the growth of intestinal bacteria species involved in immunity development, reduced the inflammatory response in the BALF, improved immune system markers, and increased pulmonary bacterial clearance, demonstrating the feasibility of such approaches in chronic lung diseases. The modulation of the immune system by diet-induced changes in gut microbiota shown in these pulmonary infected mice is also the connecting element that lead the protection against allergic airway inflammation induced by dietary fiber [182]. Thus, increases in dietary fiber induce changes in the circulating levels of short chain fatty acids produced by gut microbiota which, in turn, enhances the generation of macrophages and dendritic cells, that will eventually be responsible for the protective effect of fiber intake. In fact, nutritional-based therapies consisting of probiotic intake are proposed for their ability to enhance the pulmonary immune response [159,183]. Interestingly, the manipulation of gut microbiota in colonized mice with human selected gut bacteria affects TGF-β signaling, through the production of short chain fatty acids [184]. The modulation of TGF-β response activates Treg cells, and this could influence inflammatory conditions. Since alterations in the immune system plays an important role in IPF, it would be interesting to analyze the effects of nutritional-based strategies that improve immune function through gut microbiota for the management of this pulmonary disease.

Dietary manipulations can also impact lung microbiota composition. For example, dietary fiber is able to modify the lung microbiome [182], potentially triggered by changes in gut microbiota via the gut-lung axis, although gut microbiota independent effects elicited by fiber, or any other bioactive food ingredient, on lung microbiota composition and/or activity should not be discarded. In this regard, the potential anti-fibrotic effect of curcumin could involve the inhibition of bacterial activity with pro-fibrotic potential [185]. Likewise, the potential role of iron on IPF could be related to the iron availability to lung bacteria, which depend on the strength of the immune system, and could influence infection [186].

## 6. Conclusions and Future Directions

Overall, the evidence presented in this review supports a role for diet and particular bioactive food ingredients in IPF, as has been previously discussed for other chronic lung diseases [187], and suggests that nutritional approaches should be considered as potential complementary therapies. The proposed role of dietary factors and microbiota is most probably bidirectional, whereby bioactive food ingredients could be influenced by gut microbiota, and at the same time, influence both gut and lung microbiota composition. Moreover, gut and lung microbiota are interconnected through the gut-lung axis, and this crosstalk could be modulated by dietary factors. Thus, it is plausible to think that changes in this host-wide network may eventually have an impact on the development of the fibrotic lung. In addition, circulating and BALF levels of certain nutrients or their derived metabolites could be used as diagnostic and prognostic biomarkers of IPF, as has already been proposed for particular lipids and vitamin D [45,84,85]. Future studies should be aimed at identifying novel IPF biomarkers associated with the diet, which might include microbiota-derived metabolites, as well as a more in-depth profiling of the lung and gut microbiome in IPF.
